# Biochemical characterization and comparison of aspartylglucosaminidases secreted in venom of the parasitoid wasps *Asobara tabida* and *Leptopilina heterotoma*

**DOI:** 10.1371/journal.pone.0181940

**Published:** 2017-07-24

**Authors:** Quentin Coulette, Séverine Lemauf, Dominique Colinet, Geneviève Prévost, Caroline Anselme, Marylène Poirié, Jean-Luc Gatti

**Affiliations:** 1 Unité “Ecologie et Dynamique des Systèmes Anthropisés” (EDYSAN, FRE 3498 CNRS-UPJV), Université de Picardie Jules Verne, Amiens, France; 2 Université Côte d’Azur, INRA, CNRS, ISA, Sophia Antipolis, France; Institute of Plant Physiology and Ecology Shanghai Institutes for Biological Sciences, CHINA

## Abstract

Aspartylglucosaminidase (AGA) is a low-abundance intracellular enzyme that plays a key role in the last stage of glycoproteins degradation, and whose deficiency leads to human aspartylglucosaminuria, a lysosomal storage disease. Surprisingly, high amounts of AGA-like proteins are secreted in the venom of two phylogenetically distant hymenopteran parasitoid wasp species, *Asobara tabida* (Braconidae) and *Leptopilina heterotoma* (Cynipidae). These venom AGAs have a similar domain organization as mammalian AGAs. They share with them key residues for autocatalysis and activity, and the mature α- and β-subunits also form an (αβ)_2_ structure in solution. Interestingly, only one of these AGAs subunits (α for AtAGA and β for LhAGA) is glycosylated instead of the two subunits for lysosomal human AGA (hAGA), and these glycosylations are partially resistant to PGNase F treatment. The two venom AGAs are secreted as fully activated enzymes, they have a similar aspartylglucosaminidase activity and are both also efficient asparaginases. Once AGAs are injected into the larvae of the *Drosophila melanogaster* host, the asparaginase activity may play a role in modulating their physiology. Altogether, our data provide new elements for a better understanding of the secretion and the role of venom AGAs as virulence factors in the parasitoid wasps’ success.

## Introduction

More than 60% of the 115,000 described hymenopteran species [1] have a parasitoid lifestyle [2,3]. They oviposit in (endoparasitoids) or on (ectoparasitoids) other arthropod hosts that they use as a source of nutrients during larval development, ultimately leading to host death. Parasitoid wasps thus play an important role in controlling field arthropod populations and are use as biological auxiliaries against agricultural pests. Oviposition of endoparasitoid eggs into insect hosts induces an immune response resulting in egg melanotic encapsulation. Wasps have thus evolved strategies to evade or overcome this defense as well as to regulate the host physiology, the most prevalent being the injection of venom containing virulence molecules at oviposition [4–7]. Recent venomics (transcriptomics/proteomics) studies suggest that endoparasitoid wasps’ taxa–even closely related species or strains–can rely on different venom protein cocktails to achieve parasitism success. In contrast, similar proteins have been identified as major venom components in phylogenetically distant species, as exemplified by the aspartylglucosaminidase (AGA) (N4-(beta-N-acetylglucosaminyl)-L-asparaginase; also named glycosylasparaginase; EC 3.5.1.26) abundant in the venom of both the braconid *Asobara tabida* (AtAGA) [8,9] and the figitid *Leptopilina heterotoma* (LhAGA) [10,11]. These species have common *Drosophila* hosts, including *Drosophila melanogaster*, for which they compete in the field, but they differ by their immuno-evasive (*A*. *tabida*) *versus* immuno-suppressive (*L*. *heterotoma*) strategy [12,13]. Interestingly, however, both species induce a transient paralysis of the *D*. *melanogaster* host larvae at oviposition [14–16].

Aspartylglucosaminidase is found from bacteria to mammals. In mammals, it is described as a ubiquitous lysosomal amidohydrolase that catalyzes the last stage of degradation of glycosylated proteins, i.e. the cleavage of the glycosidic bond between the sugar chain and the side chain of L-asparagine [17,18]. Its functional deficiency in humans is responsible for a lysosomal storage disease, aspartyl-glucosaminuria (AGU, OMIM entry 208400), that leads to mental retardation [19]. Human AGA is produced as a single protein chain that undergoes self-cleavage to generate an α- and a β-subunit. The mature enzyme occurs as a heterotetramer of two α- (~25-kDa) and two β- (~18-kDa) subunits, whose active residue, a threonine, is exposed at the N-terminus of the β-subunits. Human AGA can also act as an asparaginase by hydrolyzing L-asparagine to L-aspartic acid and ammonia [20–23].

Here, we describe and compare the molecular features and biochemical activities of AGAs secreted in the venom of *A*. *tabida* and *L*. *heterotoma*. We notably demonstrate that they have an efficient asparaginase activity in addition to being aspartylglucosaminidases, which brings novel hypotheses regarding their potential role in wasps’ parasitism success.

## Materials and methods

### Biological material

The *L*. *heterotoma* Gotheron and *A*. *tabida* A1 strains originate from populations collected in Gotheron and Sainte-Foy-les-Lyon (France), respectively. They were reared at 25°C on a susceptible *D*. *melanogaster* strain (Gif stock 1333) and adult individuals were kept at 18°C with water and honey. All experiments were performed with 3- to 7-day-old parasitoid females.

### Venom collection

Venom was collected by dissecting either the venom reservoir (*L*. *heterotoma*) or the multi-lobed venom gland (since *A*. *tabida* reservoir contains low amounts of venom) and dilacerating tissues in Ringer’s saline (KCl 182 mM; NaCl 46 mM; CaCl_2_ 3 mM; Tris-HCl 10 mM) supplemented with a protease inhibitor cocktail (Sigma). Venom extracts were then centrifuged for 5 min at 5,000 g to remove residual tissues and cellular debris and kept on ice or stored at -20°C until use.

### AGA sequences analysis

The cDNA sequence of *A*. *tabida* venom AGA (ACX94224) was previously reported [8,9]. *L*. *heterotoma* venom AGA (KP888635) was identified from venom apparatus transcriptomics data [10]. The *A*. *tabida* (AtAGA), *L*. *heterotoma* (LhAGA), and *Homo sapiens* (hAGA, P20933) predicted AGA protein sequences were aligned with MAFFT [24]. Signal peptide and N-glycosylation sites prediction were performed with SignalP (http://www.cbs.dtu.dk/services/) and NetNGlyc (http://www.cbs.dtu.dk/services/NetNGlyc/), respectively. Pfam (http://pfam.janelia.org/) was used to search for protein domains. Protein disulfide bonds were predicted using DIpro (http://scratch.proteomics.ics.uci.edu/).

Molecular modeling of mature AtAGA and LhAGA was done with the Phyre server using default parameters (http://www.sbg.bio.ic.ac.uk/phyre2/) [25]. For both proteins, the model with highest confidence (100%) and coverage (76% and 75% for AtAGA and LhAGA, respectively) was obtained using the crystal structure with PDB code 1P4V as template. Model quality was assessed by the QMEAN server (http://swissmodel.expasy.org/qmean/) [26]. Briefly, QMEAN score is a global reliability score with values ranging between 0 (lower accuracy) and 1 (greater accuracy). The associated Z-score relates this QMEAN score to the scores of a non-redundant set of high-resolution X-ray structures of similar size with ideal values being close to 0. Visualization of AtAGA and LhAGA model structures, superposition with the hAGA crystal structure (PDB code: 1APY) [27], and root mean square deviations were obtained using PyMOL v0.99 (http://www.pymol.org).

### SDS-polyacrylamide gel electrophoresis

Proteins were separated on 12.5% or 15% SDS-PAGE. For reducing and non-reducing conditions, samples were mixed with Laemmli buffer [28] in presence or absence of 2.5% β–mercaptoethanol, respectively. For reducing SDS-PAGE, samples were heated for 10 min at 95°C before loading. Gels were silver stained according to Morrissey [29]. Average molecular weights of AGA subunits were estimated from four independent SDS-PAGE experiments.

### Immunoblotting

Following SDS-PAGE electrophoresis, proteins were transferred onto a nitrocellulose membrane [30]. The membrane was blocked with 2% non-fat milk in TBS-Tween and incubated overnight at 4°C with one or a combination of the following primary rabbit polyclonal antibodies: anti-hAGA, raised against the α-subunit of the human AGA (anti-24; Generous gift from the Department of Medical Genetics, National Public Health Institute, Helsinki, Finland); anti-P30 and anti-P18, raised against the α- and β-subunits of AtAGA, respectively [9]; anti-LhAGA produced against a mix of synthetic peptides, one designed from the α-subunit and the other from the β-subunit of LhAGA. Antibodies directed against AtAGA and LhAGA were species-specific while the anti-hAGA cross-reacted with both AtAGA and LhAGA. Primary antibodies were diluted at 1:1,000 except for anti-LhAGA (1:5,000). Following washing with TBS-Tween, membranes were incubated 2 hrs at room temperature with a goat anti-rabbit IgG conjugated to horseradish peroxidase (A6154 Sigma; 1:25,000).

For the lectin assay, Western blots were blocked with 3% BSA in TBS-Tween and incubated overnight with 2 μg/mL of different biotinylated lectins (Con A, DBA, PNA, RCA_120_, SBA, UEA I, WGA; Kit I, Vector Laboratories). After washing in TBS-Tween, blots were incubated with Streptavidin-peroxidase (Sigma; 1:2,000 in TBS-Tween 3% BSA). Reactive bands were detected using a chemiluminescent substrate (Immobilon Western, Millipore) and imaged with a cooled digital camera.

### N-deglycosylation assay

Venom was collected in 10 mM sodium phosphate buffer (pH 7.5) and supplemented with 0.2% SDS and 0.5% β-mercaptoethanol. Samples were heated at 95°C for 10 min, cooled on ice and incubated overnight at 37°C with increasing amounts (from 6.25 to 100 U) of PNGase F (Biolabs, UK). Reaction was stopped by heating at 95°C for 5 min. Carbohydrate removal was visualized by the migration shift of protein bands on SDS-PAGE after silver staining and immunoblotting with anti-AGA antibodies or by the presence/absence of signal with the biotinylated Con A. Specificity of the Con A binding was demonstrated by signal loss in presence of 200 mM of methyl-α-D-glucopyranoside.

### Enzyme activity assays

Aspartylglucosaminidase activity was measured at room temperature using the fluorogenic substrate AspAMC [L-Aspartic acid β-(7-amido-4-methylcoumarin)] (A1057, Sigma) in 50 mM Tris-HCl buffer (stock solution: 10 mM in ethylene glycol) [31]. The optimum pH for activity was assessed using the same buffer at a 4–10 pH range, with 0.5 mM AspAMC and venom extracts from 7 venom apparatus per assay. V_max_ and K_m_ were identified using 0.03, 0.06, 0.09, 0.18, 0.27 mM substrate concentrations, and 1.4 μg and 0.3 μg of FPLC-purified AtAGA and LhAGA, respectively. The release of the fluorescent 7-amino-4-methylcoumarin was measured continuously after substrate addition (Xenius XM fluorometer, SAFAS; λ_exc_ 350 nm and λ_em_ 450 nm), and the initial speed of the reaction was estimated during the first hour (linear part of the curve). As venom extracts or AGA-containing FPLC fractions had a similar activity whether freshly prepared or stored at -20°C, frozen and fresh samples were used indifferently.

Since L-asparagine inhibits the aspartylglucosaminidase activity of human AGA [21], we tested whether it acted similarly on *A*. *tabida* and *L*. *heterotoma* venom. The AspAMC assay was performed using 0.5 mM substrate and venom extracts from 2 venom apparatus, with various concentrations of L-asparagine (0.5, 1, 2 and 5 mM) or 5 mM glutamine, an amino acid with similar properties to L-asparagine, to test for enzyme specificity.

In order to determine the L-asparaginase V_max_ and K_m_ of the FPLC-purified AtAGA and LhAGA, we used an enzyme-coupled assay [32]. L-asparaginase catalyzes the hydrolysis of L-asparagine to L-aspartate and glutamic oxaloacetic transaminase (G2751, Sigma) subsequently catalyzes the transamination of L-aspartate and α-ketoglutarate to oxaloacetate and L-glutamate. Oxaloacetate is then reduced to malate in the presence of malic dehydrogenase (M2634, Sigma) with oxidation of NADH to NAD^+^. The change over time in absorbance of NADH at 340 nm, monitored on a microplate reader (Spectramax plus, Molecular Devices), thus allows direct evaluation of the sample asparaginase activity. All assays were done in Tris buffered saline at pH 7.4, following a first calibration with increasing amounts of the *E*. *coli* asparaginase (100–300 units/mg; Sigma). For V_max_/K_m_ determination, L-asparagine concentrations were 0.03, 0.06, 0.09, 0.17, 0.26 mM and quantities of FPLC-purified AtAGA and LhAGA were 0.9 μg and 0.7 μg, respectively. The initial speed was estimated during the first hour of the reaction (linear part of the curve).

In both aspartylglucosaminidase and asparaginase assays, V_max_ and K_m_ were obtained from Michaelis-Menten Substrate-Velocity Curves fitted using the GraphPad Prism module (V5; www.graphpad.com), and average values were calculated from two independent experiments. Similar values were found using the nonlinear least-squares fitting (Microsoft Excel Solver pack) [33].

### FPLC purification of AGA

Size fractionation of venom proteins was carried out by fast protein liquid chromatography (FPLC; ÄKTA microsystem, GE Healthcare) using a Superdex 75 column (Superdex 75 PC 3.2/30; GE Healthcare) and Ringer’s saline as a buffer, with a 50 μl/min flow. The column elution time was calibrated using native aprotinin (6.5 kDa), cytochrome C (12.4 kDa), carbonic anhydrase (29 kDa), albumin (66 kDa), and blue dextran (2,200 kDa) (Sigma Kit). For each run, 60 μl of venom extracts obtained from 50 to 75 venom apparatus were injected. Absorbance was followed at 280 nm and fractions of 50 μL were collected every minute in non-binding microplate (Greiner Bio-One). An aliquot of each collected fraction was analyzed by SDS-PAGE under reducing conditions, and the AGA-peak fractions were determined by immunoblotting and enzymatic activity assay. The fraction containing purified AGA (AGA representing more than 60% of total proteins of the fraction) was then used for estimation of kinetic constants and cross-linking experiments. Because purified AGAs were in limited quantities, we estimated AGA α- and β-subunits amounts using a silver stained gel method. Briefly, increasing volumes of the purified fractions were separated by SDS-PAGE under reducing conditions together with known amounts of BSA. Band intensities were obtained from digitalized images of the silver-stained gels using ImageJ software (http://imagej.nih.gov/ij/).

### Cross-linking with glutaraldehyde

To analyze the oligomerization state of purified native AtAGA and LhAGA, subunits were cross-linked by increasing concentrations of glutaraldehyde before separation by SDS-PAGE under reducing conditions. Aliquots (5 μl) of the AGA FPLC fraction were incubated overnight at 4°C in Ringer’s saline without (control) or with increasing concentrations of glutaraldehyde.

### Extracellular autoactivation

To test for AGA extracellular auto-cleavage and auto-activation following venom collection, extracts from 140 venom apparatus were pooled and incubated at room temperature in 280 μl of Ringer’s saline. Aliquots were removed at different times (0, 2, 4, 10, 24, 48 and 120 hrs), frozen and stored at -20°C until assayed for AGA activity, and then analyzed by SDS-PAGE under reducing conditions followed by silver staining.

## Results

### Sequence analysis and homology modeling of *A*. *tabida* and *L*. *heterotoma* venom AGAs

**Fig 1:**
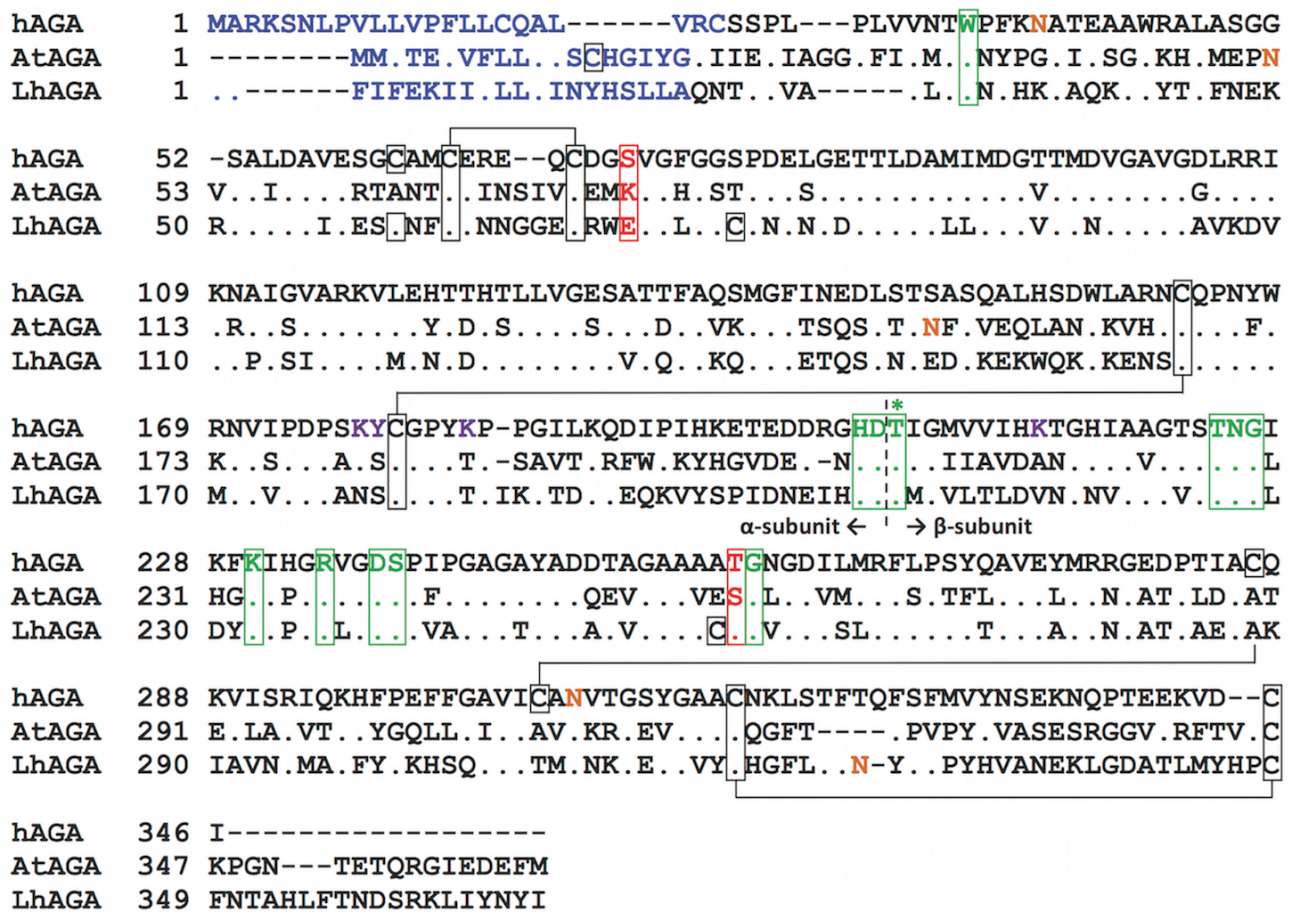
Alignment of AGA sequences. Translated cDNA sequences of *A*. *tabida* (AtAGA; ACX94224) and *L*. *heterotoma* venom AGAs (LhAGA; KP888635) aligned with the sequence of human AGA (hAGA; P20933). Residues identical with hAGA are indicated by a dot. Signal peptide amino acids are in blue. Conserved residues important for structure or activity are highlighted in green, with an asterisk indicating the active-site threonine (hAGA T206). Important hAGA residues not conserved in AtAGA and/or LhAGA are highlighted in red. Cysteine residues are framed by a black box and those involved in disulfide bonds are connected with black solid lines. Potential N-glycosylation sites are in brown (hAGA N38 and N308, AtAGA N52 and N153, LhAGA N326). Residues important for hAGA phosphorylation are in purple (K177, Y178, K183 and K214).

The predicted protein sequences of *A*. *tabida* and *L*. *heterotoma* venom AGAs (NCBI: AtAGA (ACX94224) [9]; LhAGA (KP888635) [10]) are 362 and 367 amino acid long, respectively. They contain an N-terminal signal peptide of 19 (AtAGA) and 21 (LhAGA) amino acids (Fig 1), followed by the pro-α-β-chain glycosylasparaginase domain. Sequence alignment shows that they are 45% identical and share 48% (AtAGA) and 44% identity (LhAGA) with the human AGA (hAGA; P20933). The most important residues for activity are conserved (Fig 1), including the hAGA T206 residue, essential for enzyme catalysis and autocatalytic activation [34] and the W34, R234 and D237 residues, involved in substrate binding [35,36].

**Fig 2:**
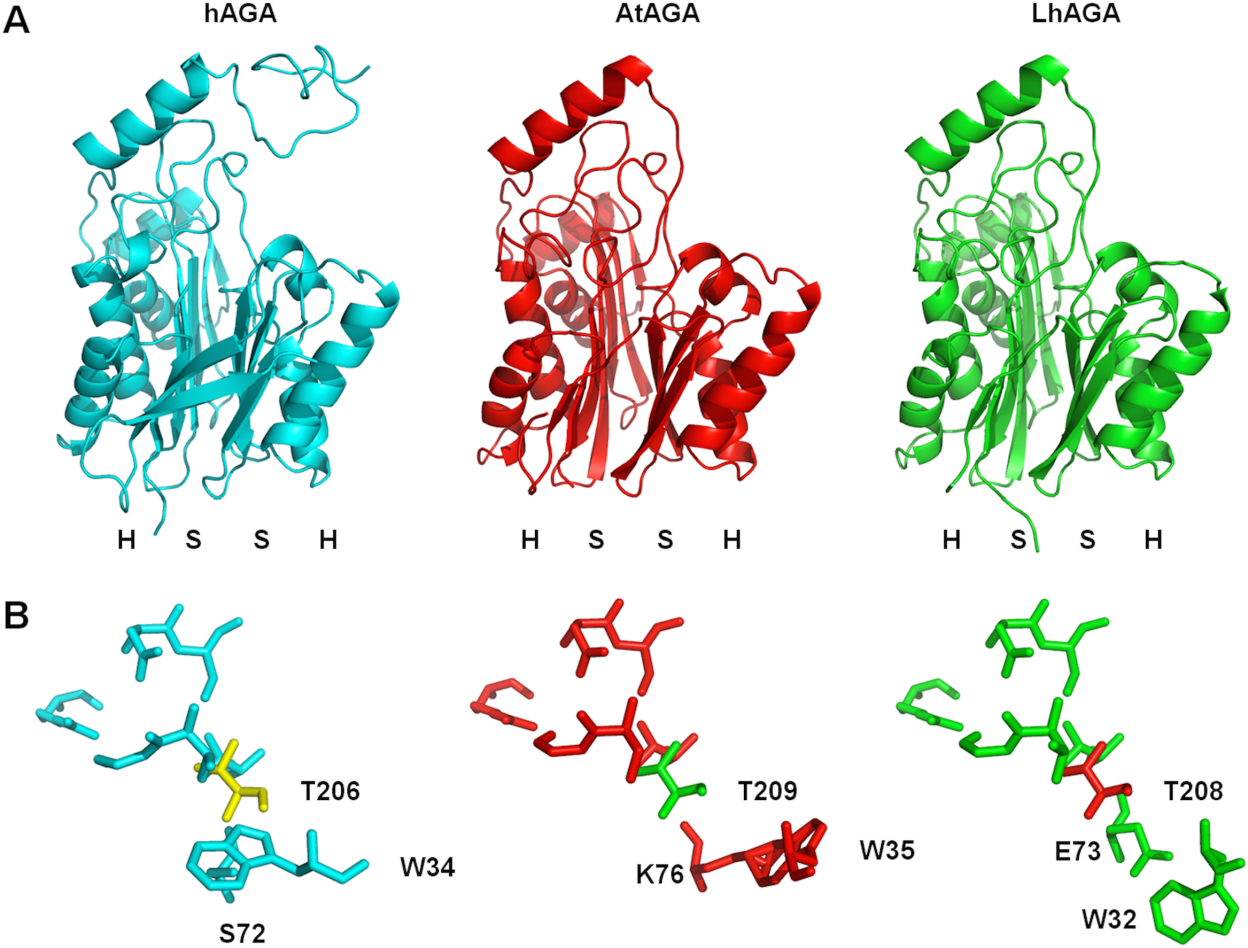
Homology modeling of AtAGA and LhAGA. A. Superposition of the active (αβ)_2_ tetramer tertiary model structure of mature AtAGA (QMEAN score = 0.694; QMEAN Z-score = -0.83; colored in red) and LhAGA (QMEAN score = 0.725; QMEAN Z-score = -0.5; colored in green) with the solved structure of human AGA (hAGA; 1APY; colored in cyan). B. Spatial geometry of some of the key catalytic and binding residues of hAGA, AtAGA and LhAGA active sites.

The predicted molecular weights of the α- and β-subunits peptide chain are 21 kDa and 16 kDa for AtAGA and 21 kDa and 17 kDa for LhAGA, respectively. Predicted model structures corresponding to amino acids 28 to 336 and 22 to 335 of AtAGA and LhAGA, respectively, were superposed with the solved human AGA structure, evidencing a strong conservation of the overall structure (root mean square deviations ranging from 0.989 to 1.011 Å for 243 to 247 α-carbon atoms) (Fig 2A). Yet, the hAGA W34 residue and the corresponding residues in AtAGA and LhAGA were predicted to differ in their spatial geometry (Fig 2B), and differences were also observed at positions 72 and 257 of the *H*. *sapiens* sequence (Fig 1). In the human AGA, the hydrogen bound formed between the polar amino acid serine or threonine at position 72 and the T206 key residue seems to be required for efficient enzymatic catalysis [34]. Surprisingly, AtAGA and LhAGA contain a lysine (polar, positively charged) and a glutamic acid (polar, negatively charged) at the equivalent 76 and 73 positions, respectively, which may weaken the formation of the hydrogen bond and affect activity. AtAGA also has a serine at position 257 while LhAGA and the human AGA both have a threonine. Interestingly, the T257S conservative substitution was shown to reduce the activity of human AGA by 30–37% [34,37], predicting a lower activity of AtAGA compared to LhAGA. Three out of the four hAGA disulfide bonds were predicted for *A*. *tabida* and *L*. *heterotoma* AGAs (Fig 1). One supplementary cysteine residue was found in AtAGA (C14) but it is in the signal peptide and thus probably lost during the maturation process. Three supplementary cysteine residues were found in LhAGA sequence (C60, C79 and C258) but due to their spatial location and orientation in the predicted structure it is unlikely that they participate in intra- or inter-subunit disulfide bonds.

### Electrophoretic profiles and immunoblotting of *A*. *tabida* and *L*. *heterotoma* venom AGAs

**Fig 3:**
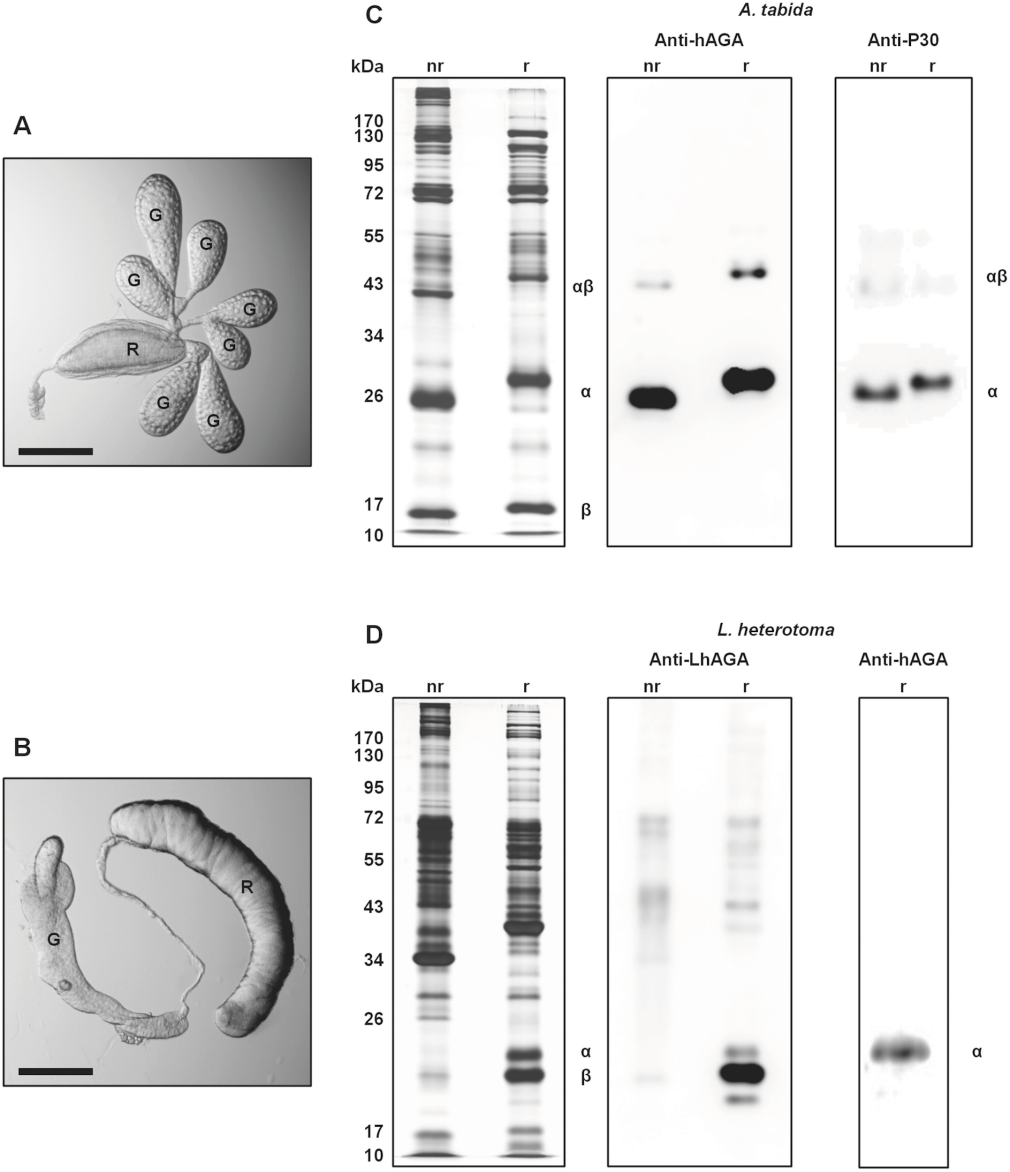
SDS-PAGE separation of *A*. *tabida* and *L*. *heterotoma* venom extracts and AGA immunostaining. A and B. *A*. *tabida* (A) and *L*. *heterotoma* (B) venom apparatus (G: gland; R: reservoir; scale bar: 0.2 mm). C and D. Electrophoretic profiles of venom extracts and immunostaining of AGA. Venom proteins from *A*. *tabida* (C) and *L*. *heterotoma* (D) were separated on 12.5% gels (2.5 venom apparatus/lane) under non-reducing conditions (nr) and reducing conditions (r). Gels were silver-stained or analyzed by western blot using the anti-hAGA, anti-P30 (*A*. *tabida* AGA) and anti-LhAGA (*L*. *heterotoma* AGA) antibodies.

Electrophoretic profiles of *A*. *tabida* and *L*. *heterotoma* venom extracted from their venom apparatus (Fig 3A and 3B) largely differ, with almost no major protein bands at the same apparent molecular weight either under reducing or non-reducing conditions (Fig 3C and 3D). On Western blots of *A*. *tabida* venom (reducing and non-reducing conditions), the anti-P30 (directed against the AtAGA α-subunit) and the anti-hAGA (directed against the human α-subunit) antibodies strongly reacted with a band at about 25–27 kDa confirming that it is the α-subunit (Fig 3C). The weak signal observed at about 40–43 kDa may correspond either to a non-active unprocessed polypeptide or to a processed αβ-heterodimeric form of AtAGA, as shown for *S*. *frugiperda* AGA [20]. Immunoblotting of *A*. *tabida* venom extracts with anti-P18 (anti-AtAGA β-subunit) led only to a faint signal at 18 kDa (see [9]). The upward shift in the migration of the two bands under reducing conditions may be due to the change in physical properties of the proteins due to disruption of disulfide bonds.

**Fig 4:**
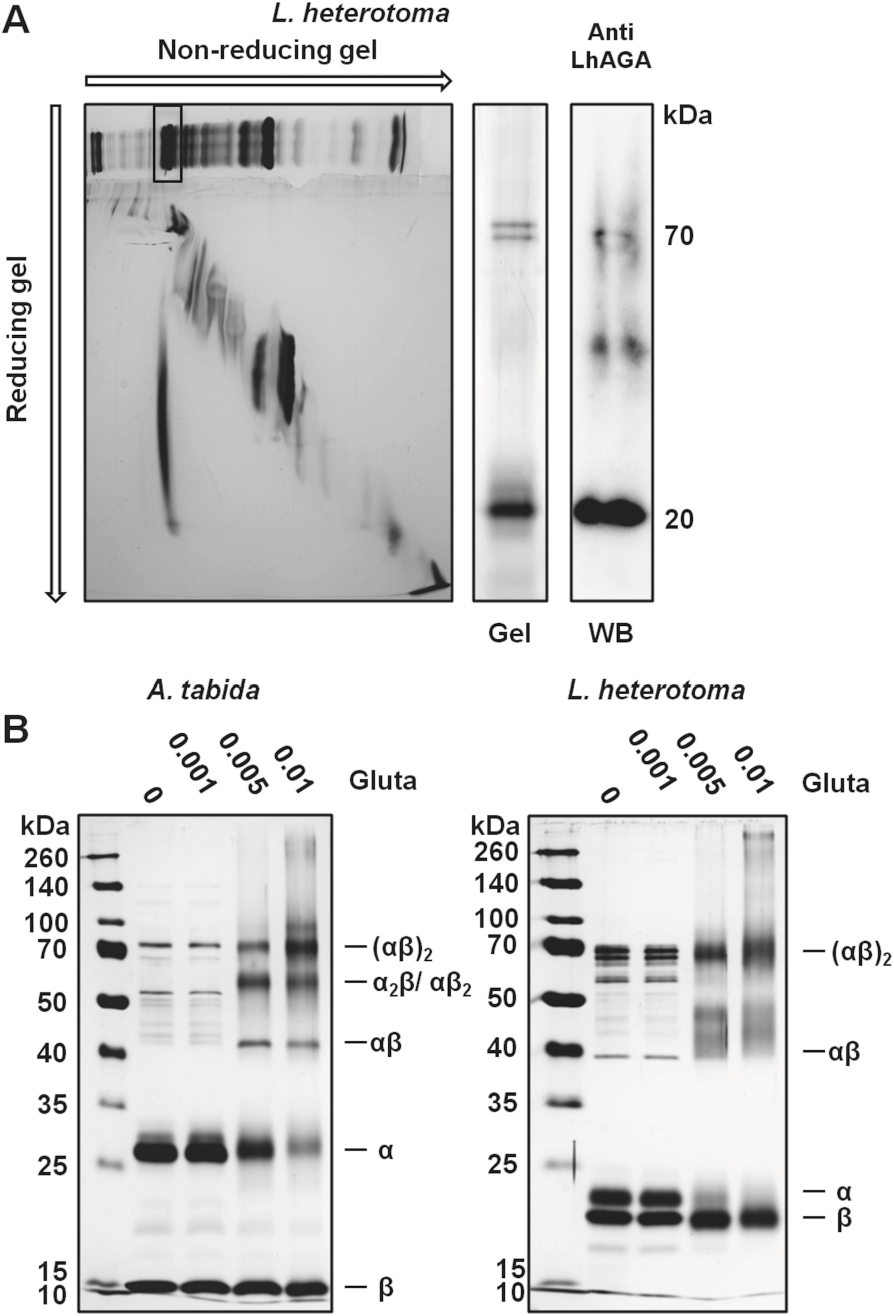
Analysis of the native conformation of AtAGA and LhAGA. A. Non-reducing/reducing electrophoretic migration of *L*. *heterotoma* venom. After venom separation on a 12.5% non-reducing gel, the full lane was excised and run under reducing conditions (gel on the left). The only band that showed a migration shift (boxed) was excised, run under reducing conditions, and silver stained (Gel) or probed with the anti-LhAGA (WB) (lanes on the right). B. Glutaraldehyde cross-linking analysis of the oligomerization state of FPLC purified native AtAGA and LhAGA (% of glutaraldehyde on top of the lane). First lane: molecular weight markers.

**Fig 5:**
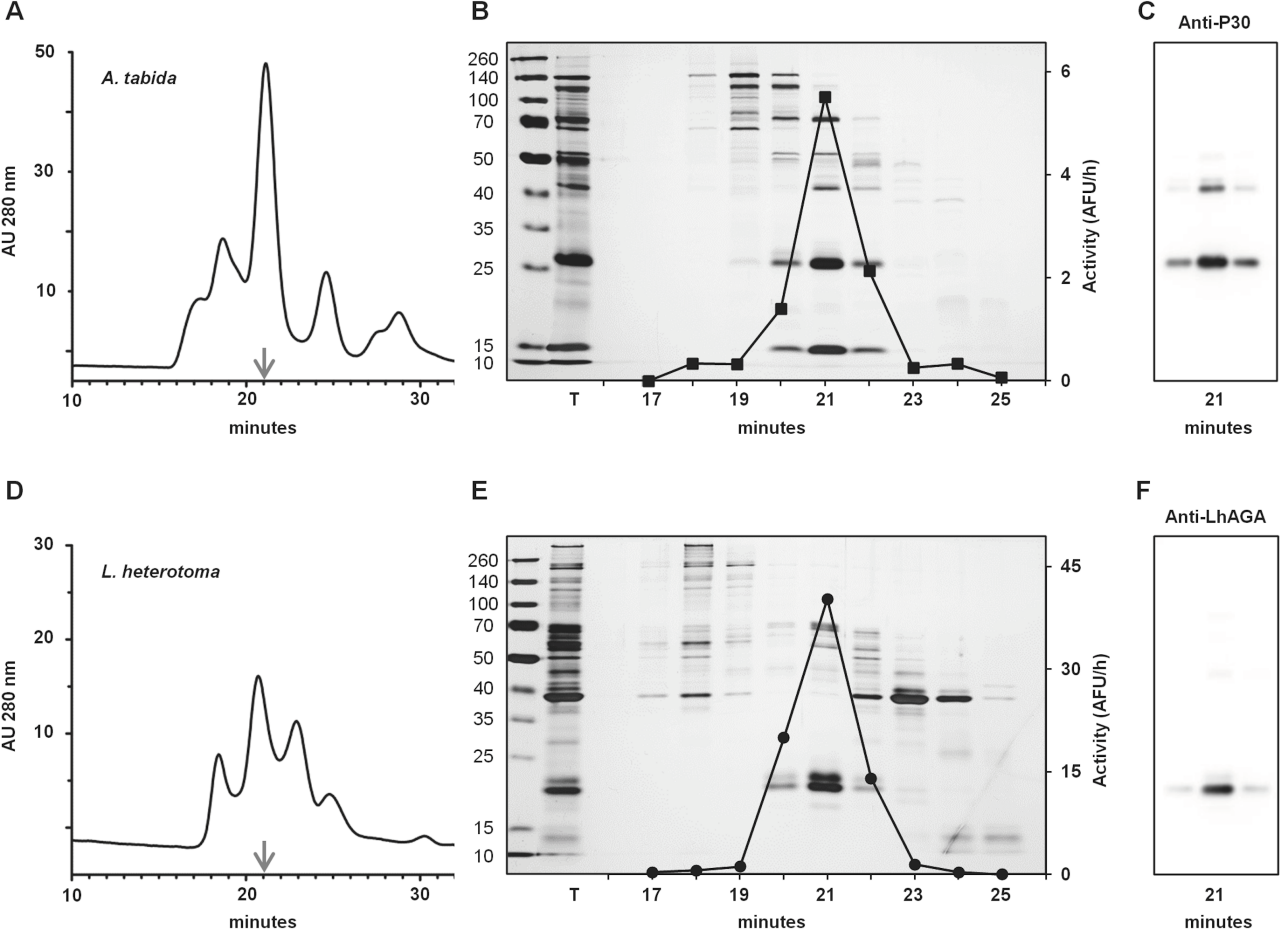
FPLC purification profiles of AtAGA and LhAGA. A and D. FPLC profiles of *A*. *tabida* (A) and *L*. *heterotoma* (D) venom extracts at 280 nm. B and E. 12.5% SDS-PAGE analysis of each FPLC fraction for *A*. *tabida* (B) and *L*. *heterotoma* (E). Lane T, total venom extract (3 venom apparatus/well). Aspartylglucosaminidase activity measured with 20 μl of each FPLC fraction is overlay on gel pictures for AtAGA (B) and LhAGA (E). C and F. Detection of AGA on western-blots of 10 μl of each FPLC fraction of *A*. *tabida* (anti-P30 antibody, C) and *L*. *heterotoma* (anti-LhAGA, F). Only AGA positive fractions are shown.

In *L*. *heterotoma* venom, the anti-hAGA antibody detected one band at about 22 kDa under reducing conditions (Fig 3D). The anti-LhAGA antibody (raised against one α and one β chain peptides) reacted weakly with the same band and strongly with a band at about 20 kDa. To ascertain the identity of these reactive bands, the equivalent 20 kDa and 22 kDa bands from the silver stained gel were analyzed by mass spectrometry (S1 Fig) and identified as the β- and α-subunit, respectively. This suggests that the anti-LhAGA antibody mainly recognizes the β-subunit. A weak staining was also observed with a 17 kDa band, very faint on the silver stained gel and thus not further analyzed, that may correspond to a degradation product of either the α- or the β-subunits. In contrast to what was observed with *A*. *tabida*, the LhAGA α- and β-subunit bands were absent from the silver-stained gel under non-reducing conditions, and no clear reaction was observed on western blots. This suggests that the LhAGA α- and β-subunits remained associated and that the epitopes were masked. We further investigated the non-reducing profile of *L*. *heterotoma* venom by running the full lane excised from the non-reducing gel under reducing conditions (Fig 4A). We observed a shift of a single band at about 70–80 kDa to a 20–25 kDa band (possibly containing both the α and β chains) that reacted strongly with the anti-LhAGA antibody, indicating that the non-reduced form may be the (αβ)_2_ form. The occurrence of a native (αβ)_2_ heterotetrameric conformation in solution was further confirmed for both AtAGA and LhAGA by: i) glutaraldehyde cross-linking experiments and ii) gel filtration FPLC. Increasing the concentration of cross-linker that maintains the tight association of subunits produced bands of increasing apparent molecular weight on reducing SDS-PAGE, with the formation of a band whose molecular weight was consistent with the size of a (αβ)_2_ heterotetramer (Fig 4B). Gel filtration purified AtAGA and LhAGA had the same elution time suggesting they have the same molecular weight (estimated at 70–80 kDa) under native conditions (Fig 5).

**Fig 6:**
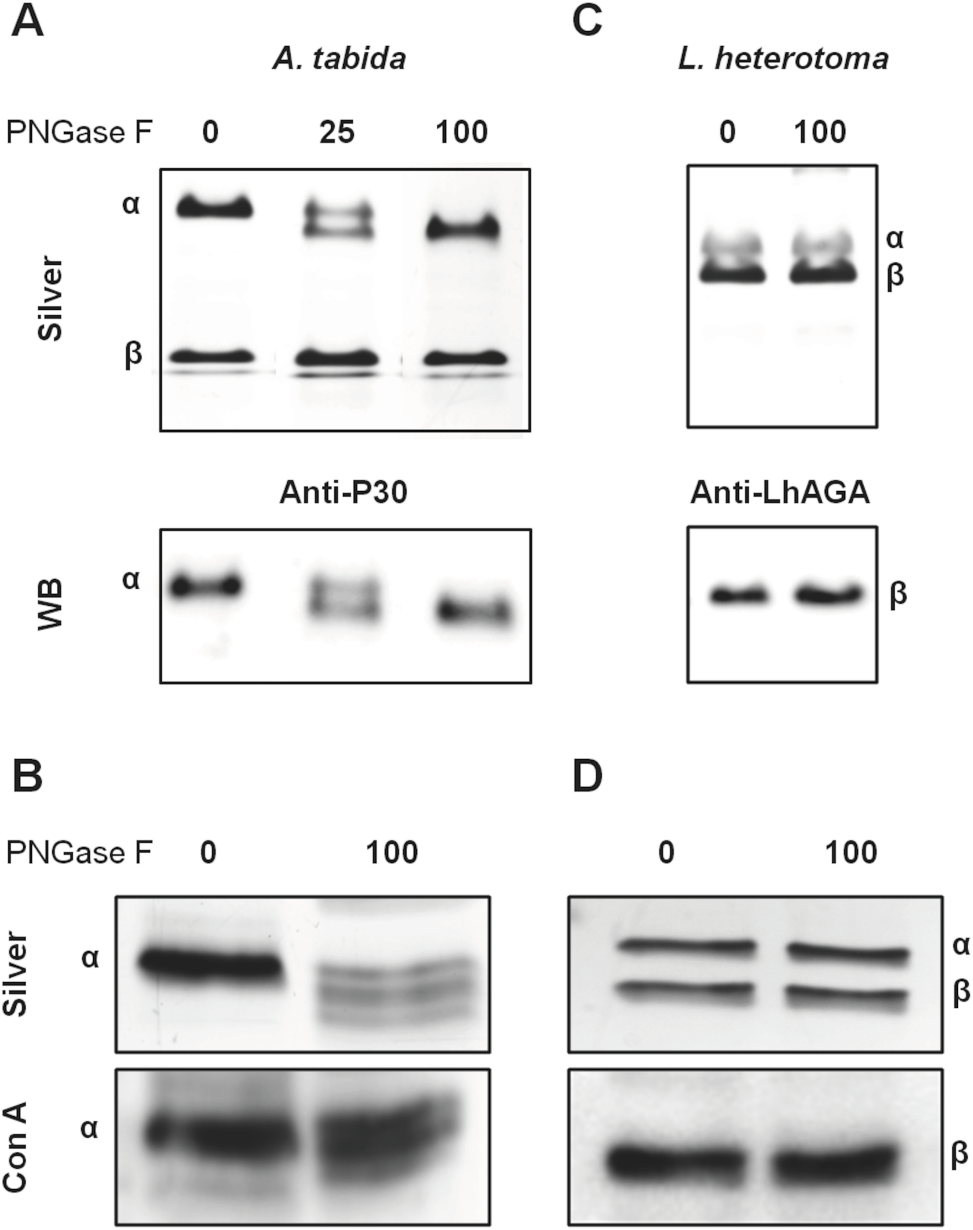
Glycosylation profile of AtAGA and LhAGA subunits. Total venom collected in PBS was heat-denatured in presence of SDS and β-mercaptoethanol and incubated overnight at 37°C in absence or presence of different quantities of PNGase F (in units). Silver stained gels (12.5% or 15%, 1/2 venom apparatus/well) and western-blots incubated with anti-P30 (*A*. *tabida*: A) or anti-LhAGA (*L*. *heterotoma*: C), or labeled with the Con A lectin (*A*. *tabida*: B; *L*. *heterotoma*: D) are shown.

The observed differences between the predicted molecular weights and those estimated from migration on SDS-PAGE (AtAGA α-subunit: 21 kDa versus 25–27 kDa; LhAGA β-subunit: 17 kDa versus 20 kDa) suggested occurrence of post-translational modifications, as reported for each α- and β-subunit of the human AGA that are N-glycosylated (N38 and N308) [38,39]. Sequences of AtAGA and LhAGA contain three potential N-glycosylation sites but only the two sites on the α-subunit (N52 and N153) of AtAGA and one site on the β-subunit (N326) of LhAGA were predicted as glycosylated by software analysis (Fig 1). N-glycosylation of AtAGA α-subunit was previously demonstrated [9]. Accordingly, treatment of *A*. *tabida* venom with PNGase F to remove N-glycosylations induced a clear mobility shift of AtAGA α-subunit from 25–27 kDa to approximately 24 kDa (Fig 6A), suggesting either an incomplete deglycosylation or occurrence of other post-translational modification. Besides, among various lectins tested on western blots of *A*. *tabida* venom, the Con A lectin strongly reacted with a band at the size of the α-subunit (27 kDa) (Fig 6B). It also reacted with the α-subunit from the FPLC-purified fraction. Although the intensity of the signal was reduced, Con A labeling was still observed on the deglycosylated α-subunit, suggesting occurrence of PGNase F-resistant glycosylation sites (Fig 6B).

The migration of the LhAGA β-subunit was not affected by the PNGase F treatment, even at high amounts and using a more resolutive 15% gel to control for the absence of molecular weight shift (Fig 6C). To ascertain whether the LhAGA β-subunit is indeed glycosylated, we tested different lectins on western blot of *L*. *heterotoma* venom. Again, a strong reaction was observed with Con A only, at 20 kDa (Fig 6D), as well as with the β-subunit of the LhAGA FPLC purified fraction, that was not affected by PNGase F treatment (Fig 6D). Yet, a more intense signal was sometimes observed for the 43 kDa band with the anti-LhAGA, suggesting partial unmasking (or better accessibility) of the epitope on the (αβ) heterodimer (or the pro-α-β-chain).

Mature AGA was reported to be produced through autocleavage/activation of a precursor form [34,37,40] and the immunoreactive bands at 43 kDa we observed in venom may correspond to the pro-α-β-chain. We thus tested whether parasitoid AGAs could undergo autocleavage /activation following venom collection. No change was observed after five days of venom incubation in the proportion of *A*. *tabida* or *L*. *heterotoma* α- and β-subunits on SDS-PAGE gels or in the aspartylglucosaminidase activity (S2 Fig), suggesting that all the AGA secreted in venom is already fully mature.

### AGAs enzymatic activities

**Fig 7:**
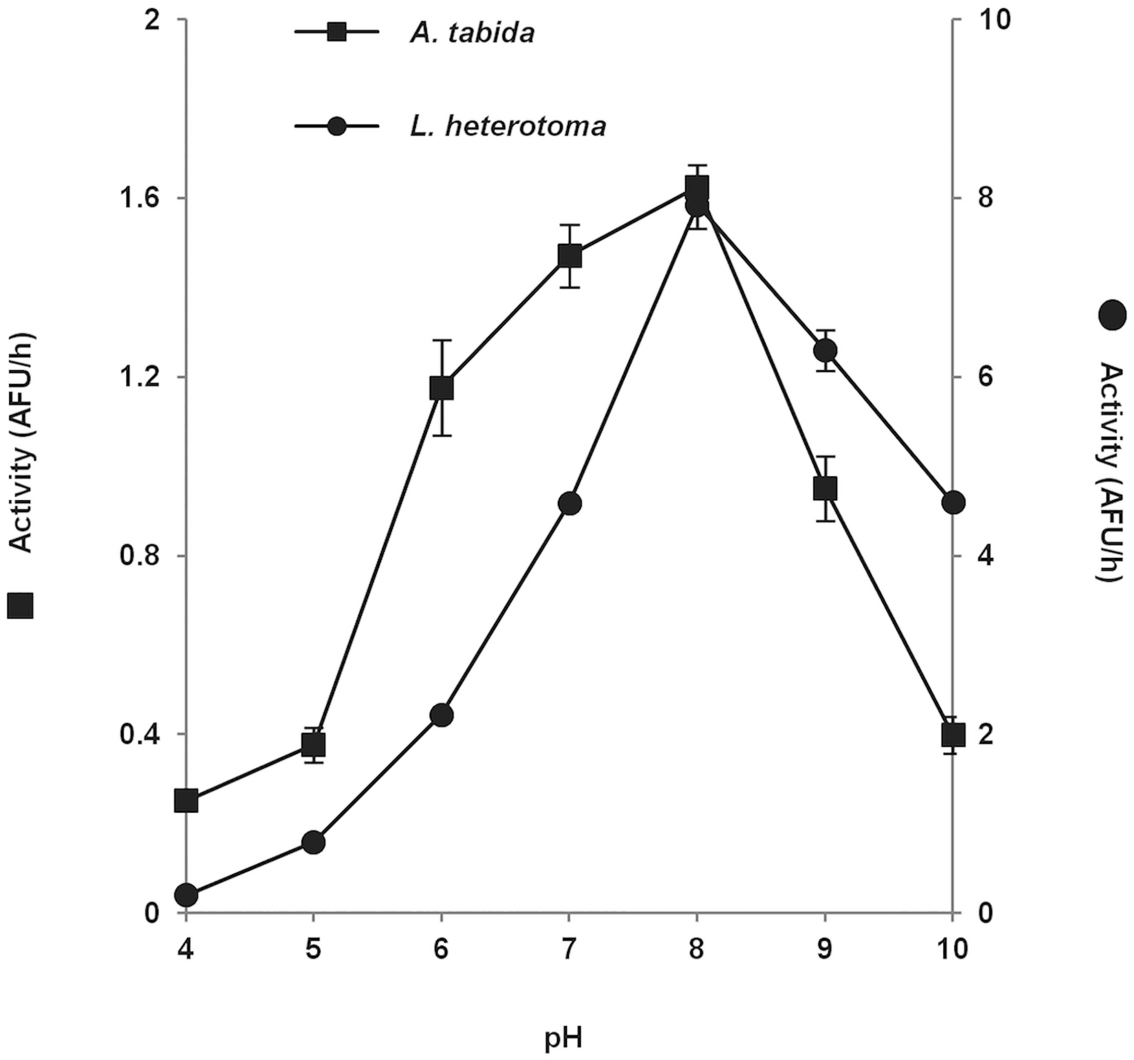
Effect of pH on AGA activity in the venom. AGA activity was measured with the AspAMC substrate in 50 mM Tris buffer at different pH. Each data point represents the mean of two separate assays with error bars showing standard deviation (SD). AFU (Arbitrary Fluorescence Units) values are indicated on the left for AtAGA and on the right for LhAGA.

The venom of both species hydrolyzed AspAMC, the substrate of a sensitive aspartylglucosaminidase assay [31]. Since only AGA immunoreactive FPLC fractions (Fig 5) hydrolyzed AspAMC, AGAs are likely responsible for all the activity detected in venom. We then defined the optimum pH of AGA activity using venom extracts (Fig 7): *A*. *tabida* AGA activity increased sharply at pH 5, then more slowly, reaching a broad optimum between pH 6 and 8 after which the activity dropped, whereas *L*. *heterotoma* AGA had a sharper activity peak centered on pH 8.

We further analyzed the catalytic properties using the FPLC purified enzymes at pH 8, optimum for the activity of both enzymes. Similar K_m_ values were obtained for AtAGA and LhAGA, but LhAGA may have a better catalytic efficiency for AspAMC, with a 10-fold higher V_max_ (Table 1).

**Table 1:** Kinetic constants of AtAGA and LhAGA for AspAMC and L-asparagine.

	AspAMC			L-Asn		
	*A*. *tabida*	*L*. *heterotoma*	*H*. sapiens*	*A*. *tabida*	*L*. *heterotoma*	*H*. sapiens**
K_m_ (μM)	74.2 ± 4.5	44.9 ± 2.2	93	4051 ± 1443	1188 ± 234	656
V_max_ (μmol/min.mg)	0.015 ± 0	0.154 ± 0.003		1.76 ± 0.53	0.75 ± 0.15	0.41
V_max_/K_m_ (μmol/min.mg)/μM	0.21 ± 0.01	3.45 ± 0.23		0.46 ± 0.03	0.63 ± 0	0.63

*(data from [31]; V_max_ not available)

**(data from [21])

As the recombinant human AGA also catalyzes the hydrolysis of the amino acid L-asparagine, which competitively inhibits the hydrolysis of aspartylglucosamine [21], we tested whether *A*. *tabida* and *L*. *heterotoma* aspartylglucosaminidase activity was inhibited by L-asparagine. We observed that AspAMC hydrolysis by venom decreased with increasing concentrations of L-asparagine, the dose-dependent inhibition reaching a maximum of about 40–45% inhibition with 2 mM of the free amino acid. In contrast, addition of 5 mM L-glutamine had no effect in these conditions, suggesting a lower affinity for this amino acid.

Finally, we confirmed that AtAGA and LhAGA have an asparaginase activity using FPLC purified enzymes. Kinetic parameters of the asparaginase activity were estimated using different L-asparagine concentrations, other conditions being unchanged. The close K_m_ and V_max_ values for AtAGA and LhAGA suggest a similar catalytic efficiency for L-asparagine which is close also to the hAGA published one (Table 1).

## Discussion

Here, we have analyzed and compared the biochemical features and enzymatic activities of secreted AGAs purified from the venom of two parasitoid wasps that belong to distant super-families: *A*. *tabida* (Ichneumonoidea) and *L*. *heterotoma* (Cynipoidea). AGA is the major protein in the venom of these species, representing 27% of the total protein content in *A*. *tabida* and 16% in *L*. *heterotoma*, based on SDS-PAGE estimation.

### Structure and secretion

In eukaryotes, AGA is described as a soluble lysosomal amidase whose genetic deficiencies in human causes aspartylglucosaminuria [36,41]. Human and other mammalian AGAs have thus been purified and characterized from different tissues and cells [42,43], as were other vertebrate AGAs [44]. Yet, to our knowledge, AGA had only been described in one insect species (Sf9 cells derived from *Spodoptera frugiperda;* Sf9AGA) [20]. All described AGAs share the same basic structure of two subunits of 19–25 kDa and 16–19 kDa, joined by non-covalent forces.

Human AGA is synthesized as an inactive single chain preproprotein precursor of about 45 kDa [35] which is translocated into the lumen of the endoplasmic reticulum (ER) [45]. After removal of the N-terminal signal peptide in the ER, two proprotein precursor chains dimerize and are glycosylated before an autocatalytic processing of each chain occurs, leading to formation of an active heterotetramer composed of two pro-α- and two pro-β-subunits [46,47] and the exposition of the threonine T206 critical for the amidase activity at the N-terminus of the β-subunit. This activation mechanism is conserved among the N-terminal nucleophile hydrolases (Ntn hydrolases) from bacteria to human [34,35,40,48]. The complex is further modified in the Golgi apparatus and then transported to the lysosomes where α- and β-subunits are carboxy-terminal proteolytically trimmed, reaching a final size of 19–25 kDa and 16–19 kDa, respectively (depending upon the studies) [49].

Sequence comparison of AtAGA, LhAGA and hAGA indicates that parasitoid venom proteins have a similar organization as lysosomal AGAs (signal peptide and glycosylasparaginase domain) and retained the threonine residue essential for enzyme autocatalytic activation and catalytic activity [34] and the triad (equivalent to hAGA W34, R234 and D237) essential for substrate binding, although they differ in their geometry [35,36]. AtAGA and LhAGA have similar predicted α- and β-subunits C-terminal disulfide bonds, which are known to be crucial in the early folding and activation of hAGA [47]. The essential hAGA stabilizing N-terminal disulfide bond [47] is also predicted for AtAGA (C66-C73) and LhAGA (C63-C70). The (αβ)_2_ structure of “native” secreted venom AGAs, suggested by the very good fit of the three-dimensional predicted structures with the hAGA solved structure, was confirmed by the 70–80 kDa molecular weight observed from gel filtration FPLC and by crosslinking experiments. Interestingly, LhAGA subunits did not dissociate on SDS-PAGE under non-reducing conditions, in contrast to AtAGA subunits. A similar resistance to SDS denaturation was previously reported for hAGA [42] and the rat AGA [50]. The reason why it is not observed for AtAGA remains unclear.

Occurrence of high amount of secreted AGA in venom is puzzling. Indeed, human lysosomal AGA is normally produced in low amounts [51,52] and AGA secretion in a specialized body fluid, albeit in low quantity, has only been reported once in mammals [53]. This suggests that specific mechanisms concur to the AGA high secretion level in parasitoid venom glands and may explain its non-lysosomal targeting.

AtAGA and LhAGA transcripts are abundant in venom tissues [9,10] compared to the residual wasp body, a pattern already observed for several potential virulence factors secreted in parasitoid venom [6]. These factors generally resemble “classical” intracellular proteins, supporting the hypothesis that the encoding genes evolved by duplication of genes involved in normal cellular processes. This mechanism may also explain the origin and evolution of venom proteins in other organisms [54,55]. *A*. *tabida* and *L*. *heterotoma* venom AGAs have thus likely evolved from an ancestral lysosomal AGA through duplication and convergent recruitment, to acquire specific features allowing overexpression and secretion. Interestingly, *in vitro* cellular overexpression of hAGA was shown to increase the proportion of AGA secreted in medium, as a prelysosomal form [38,56]. A similar process may occur for parasitoid secreted AGA: once treated with PNGase F, AtAGA and LhAGA α- and β-subunits have an estimated molecular weight close to the one predicted *in silico*, suggesting they are not trimmed at the carboxyterminal end (the predicted C-term peptide of LhAGA α-subunits was indeed obtained by mass spectrometry; S1 Fig), a process that occurs during the transport of hAGA to lysosomes.

Both α- and β-subunits of hAGA contain a single N-glycosylated site holding high-mannose-type oligosaccharides [38]. Yet, in agreement with software prediction, only AtAGA α- and LhAGA β-subunits appear N-glycosylated, according to lectin binding. Interestingly, the Sf9 AGA also has a single potential glycosylation site located on the α-subunit [20]. After PNGase F treatment, AtAGA α-subunit, but not LhAGA β-subunit, showed a clear shift in mobility, but both enzymes retained the Con A labeling. PNGase F usually cleaves all asparagine-linked oligosaccharides, whether complex, hybrid, or high mannose, unless the core contains an α(1→3)-fucose. As arthropods do not produce complex glycans [57], AtAGA and LhAGA glycosylation could be either high mannose or pauci-mannose (man-3) oligosaccharides. Thus, although we cannot exclude that one of the two potential N-glycosylation sites of AtAGA α-subunit is occupied by a modified glycan with an α(1→3)-fucose, it may also be O-glycosylated. The presence of a modified glycan or an O-glycosylation could also explain the resistance of LhAGA β-subunit to PNGase F on its sole glycosylation site. The role of hAGA glycosylations / phosphorylations on folding, activation, targeting and secretion has been assessed by *in vitro* mutagenesis and transient expression of mutant polypeptides in COS cells [38]. Results showed that glycosylation of only one subunit was sufficient for lysosomal transport and normal processing, unglycosylated enzymes remaining trapped in the ER and being secreted in the medium. Glycosylation of the β-subunit was more important than that of the α-subunit for folding, stability and transport. Thus, although the absence of glycosylation on one of the subunits may help for secretion of AtAGA and LhAGA, it is likely insufficient to explain their high secretion level.

In hAGA, three lysine residues (K177, K183 and K214) and one tyrosine residue (Y178) are necessary for proper phosphorylation of the oligosaccharides, and thus the binding to mannose 6-phosphate receptors (MPRs) [58] which is responsible for acid hydrolases lysosomal targeting in mammals [59,60]. Interestingly, the non-conservative substitution K214A in hAGA decreases the lysosomal targeting efficiency by 70%, and it is correlated with enhanced secretion [58]. In insects, the existence of a MPR-dependent pathway is unclear. Notably, the single MPR orthologue identified in *D*. *melanogaster*, named lysosomal enzyme receptor protein (LERP) [61], lacks the residues critical for mannose 6-phosphate binding and it contributes only moderately to lysosomal enzyme targeting [62]. This may explain why two of the key lysine residues (K183 and K214), and the tyrosine one, are not conserved in AtAGA and LhAGA. Besides, the K214 lysine and the tyrosine residues do not seem conserved either in other invertebrates [9]. Understanding how AtAGA and LhAGA escape the lysosomal pathway thus requires further investigations. The identification of the parasitoids’ “lysosomal” AGA encoding gene(s) for instance could allow identifying the precise molecular features and mechanisms sustaining AGA overexpression in venom and their secretion.

### Enzymatic activity

In addition to the expected aspartylglucosaminidase activity, both AtAGA and LhAGA exhibited asparaginase activity, similarly to the human and *S*. *frugiperda* AGAs. Although the optimum pH range for aspartylglucosaminidase activity was broader for AtAGA than for LhAGA, both enzymes had a maximum activity around neutrality. *S*. *frugiperda* AGA also has a broad optimum pH range (5 to 9) [20], like mammalian AGAs [34,42,63] except rat and mouse enzymes whose optimum pH is 7 to 9 [42]. Thus, although AtAGA and to a lesser extent LhAGA could be active in the lysosome acidic environment, they will have their maximum activity in the neutral *Drosophila* hemolymph environment where they are injected by the female parasitoid. We found that LhAGA has a 10-fold higher activity for AspAMC than AtAGA, which may partly be explained by the AtAGA T257S substitution [34,37] and/or by the AtAGA reduced stability. In contrast, the two purified enzymes have a similar affinity for AspAMC, in the same range as the purified human AGA (hAGA K_m_ = 93 μM at pH 7.5; similar to the K_m_ for the natural substrate GlcNAc-Asn) [31,64].

Human recombinant AGA has less affinity for L-asparagine and a lower conversion activity compared to aspartylglucosamine [21,31]. AtAGA and LhAGA also have less affinity for L-asparagine than AspAMC, similarly to Sf9AGA (K_m_ = 3.0 mM for L-asparagine compared to K_m_ = 0.9 mM for GlcNAc-Asn) [20]. Yet, insect AGAs affinity for L-asparagine is similar to that of the human asparaginase (hASNase-3; K_m_ = 2 mM at pH 7.5) [65], and they have the same catalytic efficiency as human AGA. Finally, we also estimated that AtAGA and LhAGA are 3- to 4-fold more active asparaginases than the *E*. *coli* asparaginase (used to calibrate the assay).

### Potential role of AtAGA and LhAGA in parasitism

Although the presence of N-aspartylglucosamine in *Drosophila* hemolymph seems unlikely, a concentration of 2 mM of L-asparagine was reported [66] suggesting that once injected in the host, AtAGA and LhAGA can mainly act as asparaginases to transform circulating L-asparagine in L-aspartate. L-aspartate has an excitatory role similar to that of glutamate accumulation and it is a neurotransmitter in some synapses [67]. It can act as a signaling molecule or a competitor of L-glutamate either in the brain or at the neuromuscular junctions. Its increase in hemolymph might thus play a role in the transient paralysis of host larvae induced by oviposition of both *A*. *tabida* and *L*. *heterotoma* [14–16], but also in blocking sensory class IV neurons essential for the cellular immune response to parasitoid infestation [68]. Alternatively, depriving *Drosophila* larvae from L-asparagine may induce cell division arrest and/or apoptosis and thus slow down development [69], which may help protect the parasitoid larvae and synchronize the host development with its own [70]. Indeed bacterial secreted asparaginases are known to mediate virulence by inhibiting the proliferation of immune cells through mechanisms that involve asparagine starvation, a property largely used to treat acute lymphoblastic leukemia [71–72]. The precise role of venom AGA in these suggested physiological mechanisms following from either aspartate production or asparagine depletion needs know to be elucidated.

In addition to their putative role in the success of wasp parasitism, an interest of venomous AGAs lies in the study of the mechanisms that led to their evolution from intracellular AGAs. Finally, understanding the structural features allowing a high secretion of active AGAs in the venom could potentially help improve the production of active AGA for pharmaceutical purposes [19,27,72].

## Supporting information

S1 Fig MS/MS identification of the AGA-immunoreactive 20- and 22-kDa bands resolved by SDS-PAGE of *L*. *heterotoma* venom. Identification by mass spectrometry was performed on 1D bands excised from SDS-PAGE that corresponded to the AGA-immunoreactive bands on western blots. Bands were treated with trypsin and peptides were extracted for MS/MS. Peptide identification was performed with the Mascot software (http://www.matrixscience.com) using the LhAGA sequence. Mascot analysis was performed with a fragment ion mass tolerance of 0.30 Da and a parent ion tolerance of 0.30 Da. Carbamidomethyl of cysteine was specified in Mascot as a fixed modification, and oxidation of methionine as a variable modification. The maximum miscleavage allowed was set to 2. Peptides identified with a p<0.05 are indicated in red.
(TIFF)

S2 Fig Following of AGA auto-cleavage and auto-activation venom collection. Pooled *A*. *tabida* and *L*. *heterotoma* venom extracts were incubated at room temperature and aliquots were analyzed by SDS-PAGE and western blots at different times (see materials and methods). No change in quantity of the α- and β-subunits (arrows) were observed by silver staining and for α-AtAGA and β-LhAGA by western blots (anti-P30 and anti-LhAGA, respectively). For each time, measured aspartylglucosaminidase activity is indicated (AspAMC in AFU/h). MW in kDa.
(TIFF)
