# Supplementary figures and images for: Biochemical characterization and comparison of aspartylglucosaminidases secreted in venom of the parasitoid wasps Asobara tabida and Leptopilina heterotoma
Source: PLoS One. 2017 Jul 24;12(7):e0181940. doi: 10.1371/journal.pone.0181940 (PMC5524358; doi:10.1371/journal.pone.0181940)

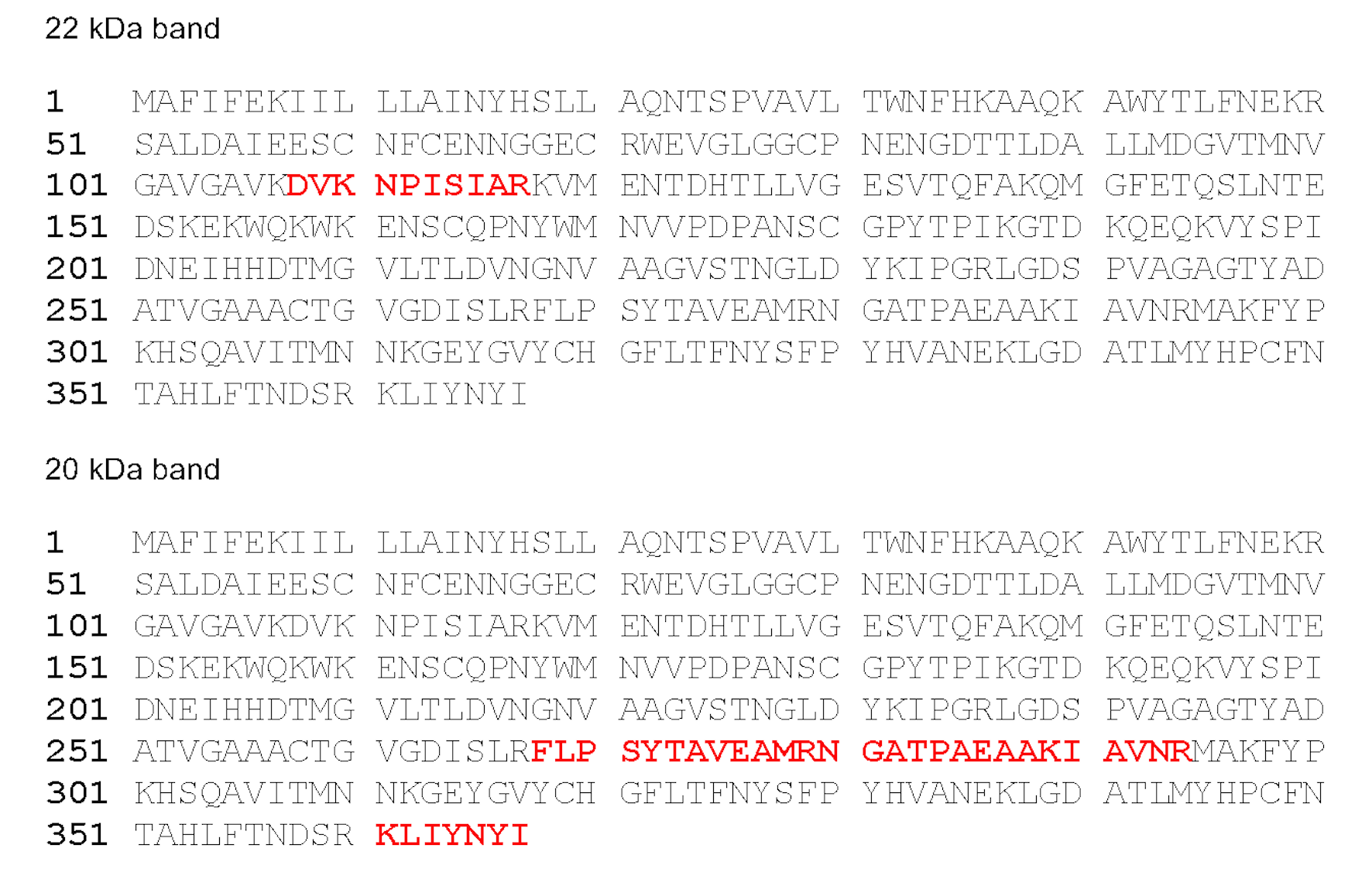

Supplement: S1 Fig — Identification by mass spectrometry was performed on 1D bands excised from SDS-PAGE that corresponded to the AGA-immunoreactive bands on western blots. Bands were treated with trypsin and peptides were extracted for MS/MS. Peptide identification was performed with the Mascot software (http://www.matrixscience.com) using the LhAGA sequence. Mascot analysis was performed with a fragment ion mass tolerance of 0.30 Da and a parent ion tolerance of 0.30 Da. Carbamidomethyl of cysteine was specified in Mascot as a fixed modification, and oxidation of methionine as a variable modification. The maximum miscleavage allowed was set to 2. Peptides identified with a p<0.05 are indicated in red. (TIFF) [file pone.0181940.s001.tiff]

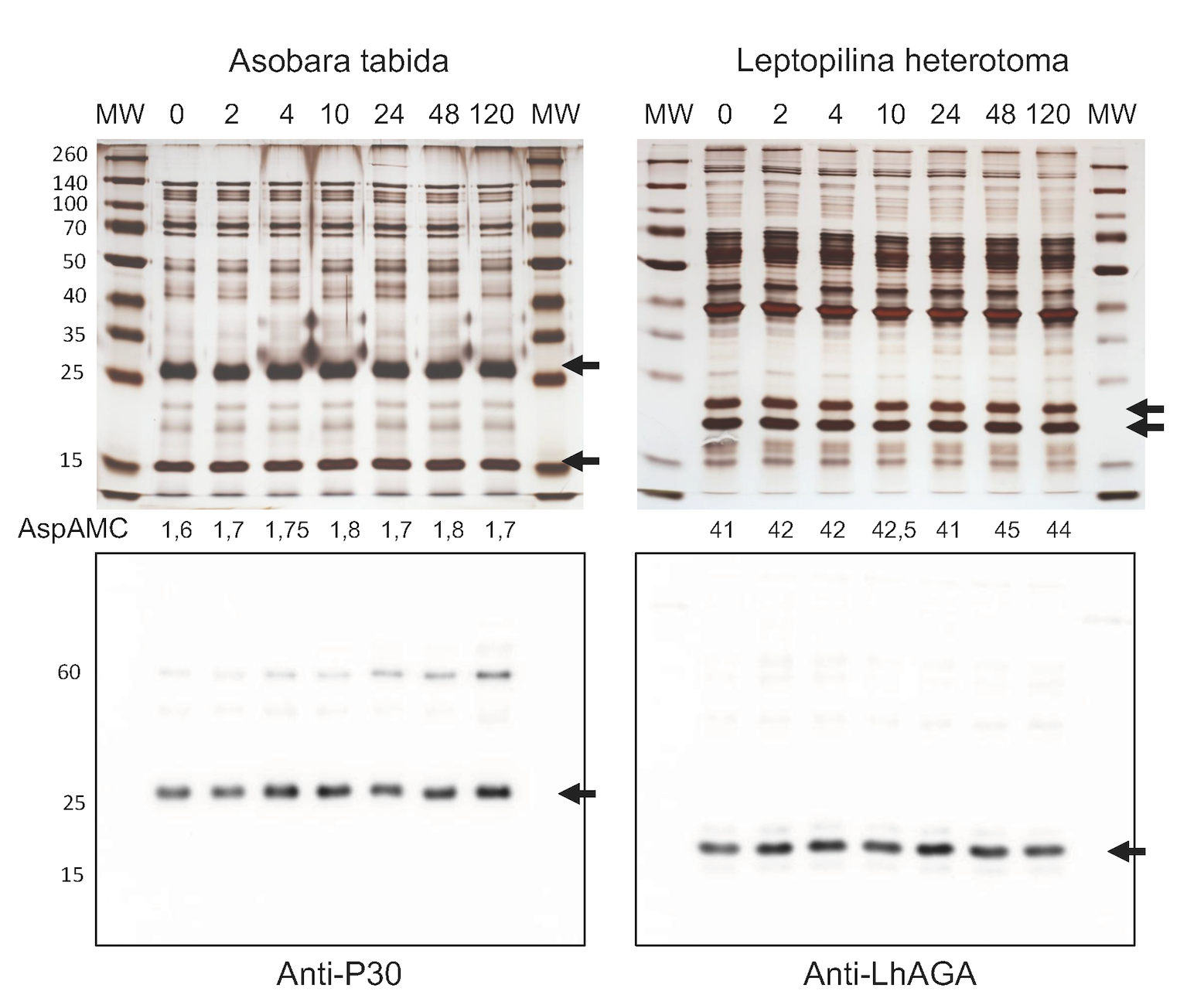

Supplement: S2 Fig — Pooled A. tabida and L. heterotoma venom extracts were incubated at room temperature and aliquots were analyzed by SDS-PAGE and western blots at different times (see materials and methods). No change in quantity of the α- and β-subunits (arrows) were observed by silver staining and for α-AtAGA and β-LhAGA by western blots (anti-P30 and anti-LhAGA, respectively). For each time, measured aspartylglucosaminidase activity is indicated (AspAMC in AFU/h). MW in kDa. (TIFF) [file pone.0181940.s002.tiff]
